# Soluble CD14 produced by bovine mammary epithelial cells modulates their response to full length LPS

**DOI:** 10.1186/s13567-024-01329-3

**Published:** 2024-06-12

**Authors:** Mégane Védrine, Florence B. Gilbert, Sarah Maman, Christophe Klopp, Christophe Gitton, Pascal Rainard, Pierre Germon

**Affiliations:** 1 ISP UMR 1282, INRAE, Université François Rabelais de Tours, Nouzilly, France; 2 SIGENAE, GenPhySE, Université de Toulouse, INRAE, INPT, ENVT, 31326 Castanet Tolosan, France; 3 SIGENAE, Genotoul Bioinfo, BioInfoMics, MIAT UR875, INRAE, Castanet Tolosan, France

**Keywords:** Mammary epithelial cell, LPS, CD14, mastitis, inflammation, *Escherichia coli*

## Abstract

Bovine mastitis remains a major disease in cattle world-wide. In the mammary gland, mammary epithelial cells (MEC) are sentinels equipped with receptors allowing them to detect and respond to the invasion by bacterial pathogens, in particular *Escherichia coli*. Lipopolysaccharide (LPS) is the major *E. coli* motif recognized by MEC through its interaction with the TLR4 receptor and the CD14 co-receptor. Previous studies have highlighted the role of soluble CD14 (sCD14) in the efficient recognition of LPS molecules possessing a full-length O-antigen (LPSS). We demonstrate here that MEC are able to secrete CD14 and are likely to contribute to the presence of sCD14 in milk. We then investigated how sCD14 modulates and is required for the response of MEC to LPSS. This study highlights the key role of sCD14 for the full activation of the Myd88-independent pathway by LPSS. We also identified several lncRNA that are activated in MEC in response to LPS, including one lncRNA showing homologies with the mir-99a-let-7c gene (MIR99AHG). Altogether, our results show that a full response to LPS by mammary epithelial cells requires sCD14 and provide detailed information on how milk sCD14 can contribute to an efficient recognition of LPS from coliform pathogens.

**Supplementary Information:**

The online version contains supplementary material available at 10.1186/s13567-024-01329-3.

## Introduction

In nature, *E. coli* is a constituent of the mammalian gut microbiota, but it is also found less commonly, in soil, water, plants, and food [1, 2]. The presence of *E. coli* in the environment could be a cause of concern because it can induce human or animal diseases. Indeed, *E. coli* is among major mammary infectious pathogens, especially in acute clinical mastitis [3].

Mastitis constitutes the main source of financial losses for dairy herds in France and worldwide [4]. These losses are mainly due to milk production decrease and treatment costs resulting in billions of euros lost per year [5].

The implication of bacterial characteristics in the severity of the infection is difficult to establish but it is known that lipopolysaccharides (LPS) represent a highly active stimulus of the innate immune system, providing an immediate immune response [6, 7]. An LPS molecule contains three regions: the lipid A, the core oligosaccharide, further divided into outer and inner part, and the O-antigen polysaccharide. LPS from the bacterial cell wall is composed of a mixture of different molecules that can vary in lipid A composition but also in polysaccharide moiety [8]. Complete LPS molecules that present an O antigen are called “smooth” (LPSS). In contrast, a “rough” (LPSR) strain expresses a core oligosaccharide but is lacking the O polysaccharide. The structure of the LPS is strongly associated with its biologic activity. Lipid A is the minimal active component required to induce an immune response [9] and most of the biological activities have been associated with this lipid moiety[10]. However, the role of the O antigen is not negligible. This has been illustrated by the different biological responses induced by LPSS or LPSR [11]. LPS is recognized by the toll-like receptor 4 (TLR4)—myeloid differentiation protein 2 (MD-2) complex that mediates lipid A recognition [12]. The action of the TLR4/MD-2 receptor complex is enhanced by the membrane-bound or soluble form of the cluster of differentiation antigen 14 (CD14) [13]. TLR4 signals depend on adaptor proteins, which operate in functional pairs: MyD88 with TIRAP (MyD88-dependant pathway), and TRIF with TRAM (TRIF-TRAM pathway). In myeloid cells, the receptor can function in two separate modes: one in which full signaling occurs in the presence of smooth or rough LPS and CD14; and one limited to MyD88-dependent signaling pathway [13]. In non-myeloid cells that do not express membrane CD14 (mCD14) the presence of soluble CD14 (sCD14) was demonstrated to be important for the recognition of LPS, as is the case with mammary epithelial cells (MEC) that constitute one of the first defense lines of the mammary gland [14]. The contribution of sCD14 to the bovine TLR4 receptor response has been previously shown in HEK293 cells transfected to express both TLR4 and MD-2 [15].

In a previous report, we investigated the bacterial genetic determinants responsible for the weak pro-inflammatory response of MEC triggered by the mastitis *E. coli* strain P4. Our results demonstrated that the presence of an O-antigen actually reduced the response of MEC to *E. coli* and to purified LPS. Indeed, purified LPSS induced a weaker response from MEC than the LPSR and the biological activity of the LPSS fraction could be restored by the addition of recombinant bovine sCD14 [16]. The differences of immune response induced by LPSR and LPSS suggested differential activation of LPS recognition pathways for these two forms of LPS in MEC.

sCD14 is present in significant amount in bovine milk and is likely to be produced by MEC as it has been detected on milk fat globules [16–18]. Yet, the production of the sCD14 by MEC has, to our knowledge, not been clearly established so far.

In the present report, we first demonstrate that primary bovine MEC are able to secrete sCD14. Using RNAseq, we then analysed in details the pathways induced in bovine MEC by the R-form and S-form of LPS from the *E. coli* P4 strain. This study aimed to decipher how MECs recognize LPS from *E. coli*, thus alerting the mammary gland of a coliform intrusion.

## Materials and methods

### Culture of bMEC and PS cells

Primary bMEC (pbMEC) and PS cells were collected from cows that were killed at the slaughterhouse of the INRAE dairy facility as part of a culling planned at the end of their 6th lactation. pbMEC were isolated from three lactating cows as previously described, cryopreserved in liquid nitrogen and used at their third passage [19]. PS cells, a bovine mammary epithelial cell line obtained in our laboratory from secretory parenchyma [14], were also used in parallel. Cells were cultured at 37 °C in 5% CO2 in growth medium (GM) made of Advanced DMEM/F-12 medium (Gibco) containing 4 ng/mL of hydrocortisone (Gibco), 2 mM of glutamine (Gibco), 20 mM of HEPES (Biowhittaker), IGF-I (Insulin-like Growth Factor; 10 ng/mL; Peprotech), FGF (Fibroblast Growth Factor; 5 ng/mL; Peprotech) and EGF (Epidermal Growth Factor; 5 ng/ mL; Sigma).

### Quantification of CD14 production by pbMEC and PS cells

For the quantification of CD14 production, pbMEC from three different lactating cows and PS cells were seeded in 25 cm^2^ flasks at a density of 1.5 × 10^5^ cells/mL in 8.5 mL of growth medium. Five hundred microliters of supernatant were collected at day 0, 1, 2, 3, 4, 7 and 8 and stored at -20 °C until use. sCD14 in the supernatant of pbMEC and PS cells was quantified by an in-house ELISA assay as described previously [16].

### Flow cytometry analysis of pbMEC and PS cells

Antibodies used in the present study were CD14-PE-AF750 (Bio-Rad, reference MCA1568P750, clone Tük4), CD45-PE (Bio-Rad, reference MCA2220PE, clone 1.11.32) and CD49F-FITC (Miltenyi Biotec, reference 130-126-008). Fixable Viability dye eFluor 450 was included in all experiments (eBioscience, reference 65–0863). Labeling was done on pbMEC and PS cells as described previously [14]. Cells were harvested after trypsin treatment (0.05% trysin-EDTA -Gibco), washed once with HBSS—10% FCS and the cell pellet was resuspended in HBSS—10% FCS. One million cells were transferred to a 1.5 mL tube, centrifuged and resuspended in FACS buffer (DPBS, 2 mM EDTA, 2% horse serum). Cells were labeled for 30 min with primary antibodies (30 min, 4 °C). After washing in FACS buffer, cells were resuspended in DPBS containing the viability dye for 30 min at 4 °C. After a final wash in FACS buffer, cells were resuspended in FACS buffer. Data were acquired with a LSR Fortessa™ X-20 Flow cytometer (Becton Dickinson) and results were analyzed with the Kaluza software (Beckman Coulter).

### Bacterial LPS

Strain P4 (serotype O32:H37) is a prototypical *E. coli* mastitis strain isolated from a bovine case of clinical mastitis [20]. Bacteria were grown routinely in Brain Heart Infusion broth (BHI) with or without shaking or on solid tryptic soy agar (TSA) medium at 37 °C. LPS was extracted from bacteria using the phenol/EDTA/triethylamine (phenol/EDTA/TEA) procedure [21] with some modifications [16]. Then, the native P4 strain LPS was fractionated by size-exclusion chromatography with a Sephacryl S-200 column (Amersham Pharmacia Biotech) to obtain LPSS. After removal of LPSS, fractions containing LPSR were subjected to preparative electrophoresis using model 491 cell from Bio-Rad as described previously [16]. LPSR and LPSS were quantified by measurement of the myristic acid content of each fraction, myristic acid being part of the conserved portion of lipid A and thus present in both LPSR and LPSS (Faculty of Medicine, Dijon) [22].

### Stimulation of PS cells with purified bacterial agonists

Stimulation assays of PS cells, at passages 12, 13 and 14, were performed as described previously [23, 24]. Briefly, PS cells were seeded at a concentration of 10^5^ cells/mL in 6-well plates in GM medium and allowed to grow to confluence. Sixteen hours before stimulation, stimulating medium (GM medium without growth factors) was added. Cells were stimulated with 250 ng/mL of purified LPS (LPSS or LPSR) diluted in fresh stimulation medium. When required, 0.5 μg/mL of recombinant bovine sCD14 was added to the medium. Recombinant bovine sCD14, with a sequence 100% homologous to nucleotides 207 to 1265 of XM_005209429.5, was purified as described previously [24]. After 5 h with purified LPS forms, total RNA was isolated from MEC. A total of 6 conditions per experiment were thus considered depending on the stimulus (CTRL (no LPS), LPSS or LPSR) and the absence or presence of sCD14 (NONE or CD14) and will be abbreviated as CTRL-NONE, CTRL-CD14, LPSS-NONE, LPSS-CD14, LPSR-NONE, LPSR-CD14. Each experiment was performed three times.

### RNA extraction

Total RNA from PS cells stimulated as described above was extracted by using the NucleoSpin RNA kit, and the residual genomic DNA was removed by using DNase digestion with RNase-free DNase (Macherey–Nagel, Germany). Obtained RNA was quantified by measuring the absorbance at 260 nm using a NanoDrop spectrophotometer (NanoDrop Technologies, North Carolina) and integrity was checked by using the Bioanalyzer system (Agilent Technologies, California). The RNA integrity value (RIN) of the samples ranged between 9 and 10.

### Illumina sequencing

Sequencing was performed at the GenoToul genomic platform (Castanet-Tolosan, France). Briefly, cDNA libraries were prepared from high quality RNA. Samples were tagged to allow subsequent identification, amplified by polymerase chain reaction (PCR) and quantified by quantitative PCR. Individual RNA-seq libraries were sequenced in duplicate at 150 bp/sequence paired-end reads using an Illumina HiSeq 3000 sequencer (Illumina, USA). Three independent stimulation experiments were performed, each with 3 conditions (unstimulated, LPSR and LPSS) with or without CD14 added.

### Reads mapping

Transcript quantification was performed using the Nextflow nf-core\rnaseq pipeline v1.4.2 [25] to map reads against the Bos taurus genome (Bos_taurus.ARS-UCD1.2.dna.toplevel.fa) and Bos_taurus.ARS-UCD1.2.99.gtf annotation. Detailed description of software versions is given in Additional file 1. Long non coding RNA (lncRNA) were identified from RNAseq data using the FEELnc pipeline [28].

### Differential expression analysis

Expression data analyses were performed using R studio (RStudio 2022.07.1). The edgeR package (version 3.36) was applied to identify statistically significant differentially-expressed genes (DEGs). The edgeR program uses probabilistic methods to determine differentially expressed genes or transcripts. The quantification file was filtered to keep only genes with at least one sample with a number of reads greater than 5. The dispersion estimation was performed using the GLMCommonDisp function. To determine DEGs following the inflammatory challenge, RNAseq control data were collected from PS cells in the absence of LPS were used. The *p*-values were adjusted using the Benjamini and Hochberg method. An adjusted *p*-value (FDR) of 0.01 was set as the threshold to select DEGs in all analyses.

### Biological interpretations of the differentially expressed genes

ReactomePA (v1.38.0), GAGE (2.44.0), SPIA (2.46.0), PathView (1.34.0) and ComplexHeatmap (2.10.0) packages were used to identify pathways and functional processes of biological importance within the list of DEGs. The DEGs (with a FDR ≤ 0.01) with their associated annotation (when present) were uploaded into ReactomePA. The significance of the pathway was measured with the FDR < 0.05 and the ratio of DEG/number of genes in the pathway. Transcription factor enrichment analysis was performed using CHEA3 [26].

## Results

### Bovine mammary epithelial cells secrete soluble CD14

**Figure 1:**
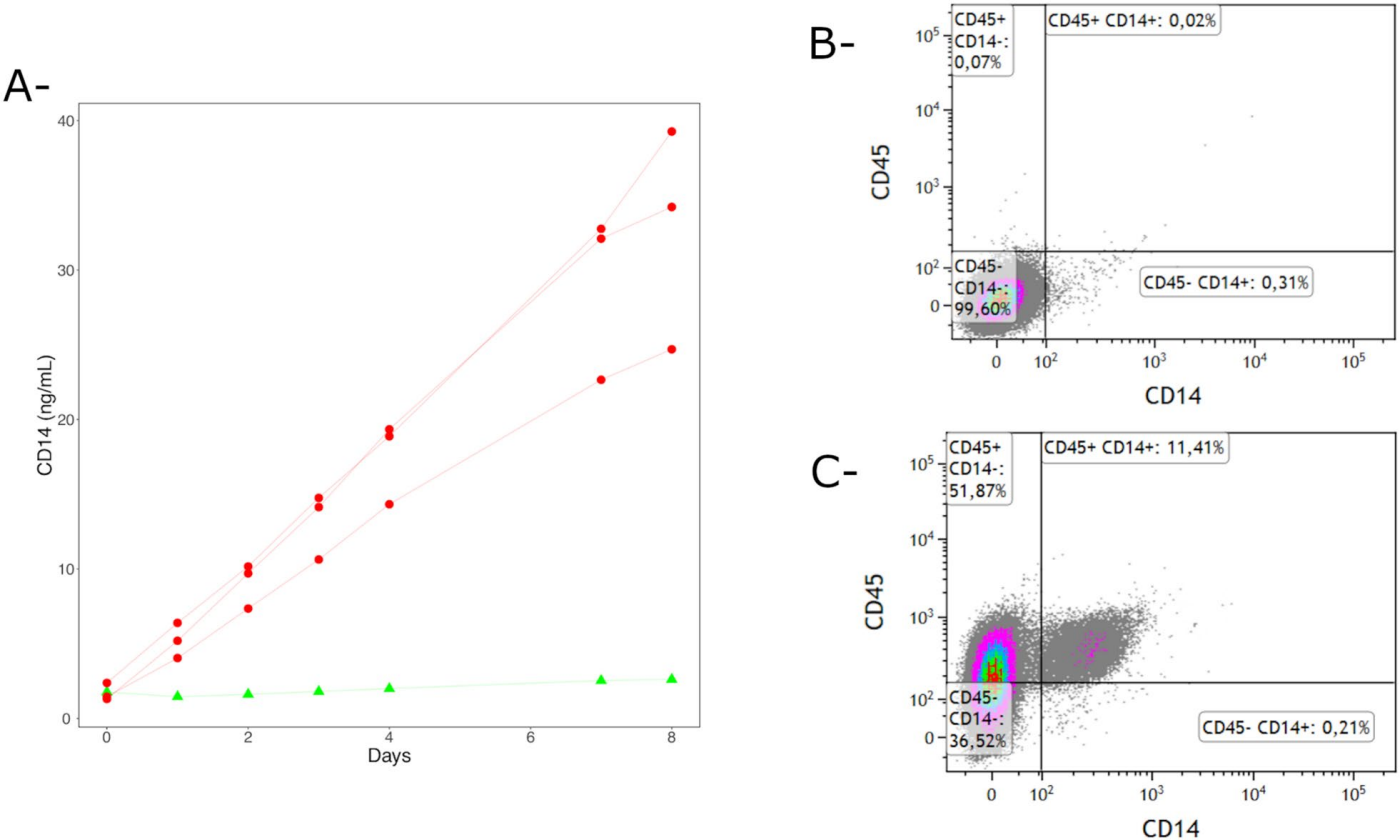
**Mammary epithelial cells secrete sCD14.** pbMEC from three different animals (red lines) and PS cells (green line) were cultured in GM medium for up to 8 days. Supernatant samples were collected at the indicated time points and used to quantify sCD14 secretion (**A**). At the end of the incubation, cells were detached and labelled to check for potential contamination by leukocytes producing sCD14. A dot-plot of one of the three batches of pbMEC used in this study is shown (**B**). Similar results were obtained with the two other batches and with PS cells. Control labeling was performed on bovine PBMC (**C**).

As a preliminary step we investigated whether bovine mammary epithelial cells are able to secrete sCD14. We thus cultivated pbMEC and the PS cell line in serum-free conditions and collected medium at different time points. The amount of sCD14 in the supernatant was quantified by ELISA. As shown in Figure 1A, significant amount of sCD14 accumulated over time in the supernatant of primary bMEC but not in that of PS cells. In order to check for a potential contamination of pbMEC with CD14 producing leukocytes, flow cytometry analysis was performed at the end of the 8-day incubation (see Additional file 2 for gating strategy). Less than 0.15% of cells were either CD45-positive and/or CD14-positive cells (Figure 1B). The few CD45-positive and/or CD14-positive dots observed were most probably due to non-specific binding as similar percentages were obtained with non-labeled cells (data not shown). This indicates that pbMEC were not contaminated with leukocytes and were likely to be the main source of sCD14 in the supernatant. Bovine peripheral blood mononuclear cells were used as positive controls for CD45 labeling (Figure 1C).

### Response of PS cells to stimulation by different forms of LPS in the presence or absence of CD14

**Figure 2:**
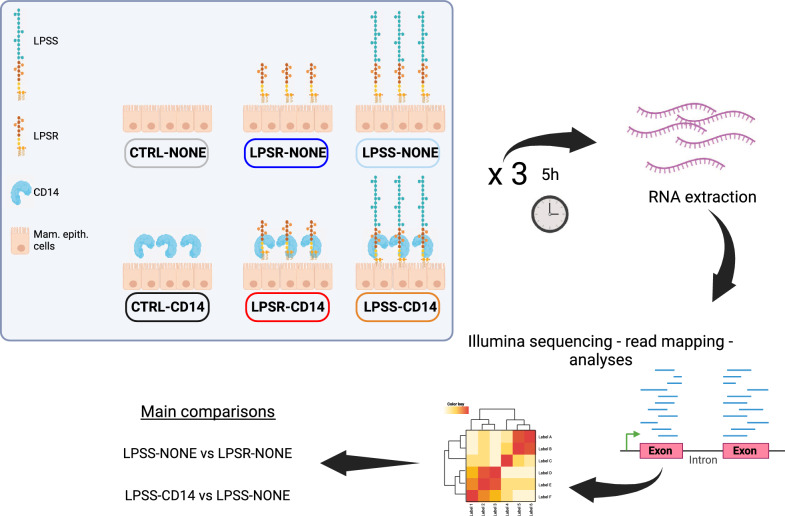
**Experimental design.** On three different days, PS cells were left unstimulated (CTRL) or stimulated with LPSS or LPSR in the presence or absence of sCD14. Five hours post-infection RNA were extracted and processed for RNA sequencing. The color code for each of the six conditions is indicated by the box surrounding the designation of the 6 conditions tested.

In order to investigate the effects of the O-antigen and sCD14 on the pro-inflammatory response of mammary epithelial cells, and avoid the potential interference due to the intrinsic production of sCD14 by pbMEC demonstrated above, we used PS cells, grown and propagated in the absence of FCS which excludes the possibility of any sCD14 originating from FCS carry-over, and stimulated them in the presence or absence of recombinant sCD14 with R- and S- forms of LPS (see Figure 2 for details in experimental design). After 5 h of contact of PS cells with the different forms of LPS, RNA was extracted and cellular responses were analyzed by RNAseq analysis. A total of 12 million paired-end reads were obtained from the transcriptome sequencing of the 18 samples analyzed. Neither LPS-fractions nor sCD14 had any effect on the number of reads. The dataset obtained with edgeR showed sample counts for 27 607 genes. Of the 27 607 genes expressed, after excluding weakly expressed genes (genes with less than 5 mapped reads in all samples), 15 700 were conserved for differential gene expression. The Multidimensional Scaling (MDS) plot and correlation plots separate the samples with and without sCD14, while the second axis separates the three independent experiments (Additional file 3). The MDS plot clearly discriminated the different conditions, supporting the hypothesis that the 15 700 genes allowed discrimination of samples.

### Global analysis of differentially expressed genes

**Figure 3:**
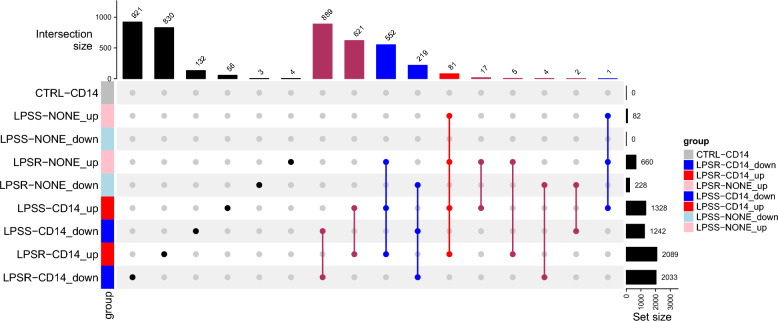
**Upset plot diagram indicating the upregulated or downregulated DEGs between the different conditions**. Control with sCD14 (CTRL-CD14); LPSS without sCD14 (LPSS-NONE); LPSS with sCD14 (LPSS-CD14); LPSR without sCD14 (LPSR-NONE); LPSR with sCD14 (LPSR-CD14) versus the condition control without sCD14 (CTRL-NONE).

In downstream analyses, genes were considered as differentially expressed between two conditions when the absolute value of the fold-change (FC) was above 1.5 and the FDR was below 0.01. Differentially expressed genes (DEG) were analyzed for each condition relative to the unstimulated cells. Using these criteria, fold-changes in expression level in all stimulation conditions were compared to unstimulated cells: a total of 4337 genes were differentially expressed in at least one condition compared to the unstimulated cells. An upset plot is depicted in Figure 3 and describes how these 4337 DEGs are distributed between the different comparisons. Among these DEG, 13 microRNA (miRNA) were deregulated in at least one condition as shown in Additional file 4.

**Figure 4:**
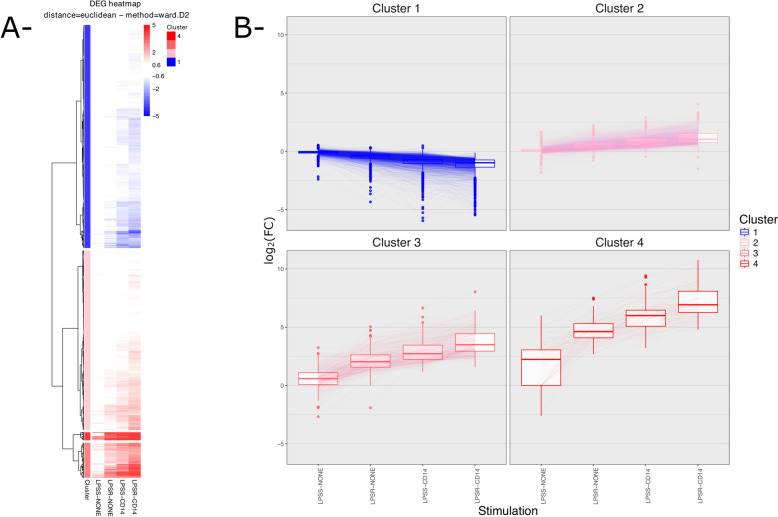
**Heatmap and parallel plots of log(FC) for all DEG.** DEG were identified using the edgeR package for the calculation of log_2_(FC) and *p*-values. (A) log_2_(FC) values against the CTRL-NONE condition were used to generate a heatmap and perform a hierarchical clustering using the pheatmap package. Four gene clusters were identified using the hclust function and are depicted on the left side of the heatmap. (B) log_2_(FC) values for genes of each of the four clusters identified in A are represented as Box-whiskers plots. Box limits indicate the range of the central 50% of the data, with a central line marking the median value. Vertical lines extend from each box to capture the range of the remaining data (between 10 and 90%). Dots placed past the line edges indicate outliers. Within a cluster, log_2_(FC) values for each gene in the four conditions are connected by thin lines.

A clustering analysis of these DEG was also performed (Figure 4). Analysis was performed on the 4265 bovine genes, out of the 4337 DEG, having an orthologue in human. Four clusters of genes could be identified with varying degrees of fold-changes. Cluster 1 genes (2135 genes) are repressed compared to unstimulated cells while the three other clusters encompass genes induced upon stimulation, cluster 2 genes (1717 genes) with the lowest FC levels and cluster 4 genes the highest FC (77 genes). Cluster 3 genes (336 genes) show intermediate levels of FC.

Overall, this analysis indicates that most DEG were observed when cells were stimulated in the presence of sCD14 with LPSR or LPSS, with more genes being deregulated when LPSR was used (Figure 3). sCD14 alone did not significantly modify the transcriptomic profile of PS cells, with no significantly differentially expressed genes identified when comparing CTRL-NONE and CTRL-CD14 conditions. LPSS alone was a weak stimulus compared to LPSR, and the addition of sCD14 markedly enhanced MEC responses highlighting the importance of sCD14 for a full response of MEC.

**Figure 5:**
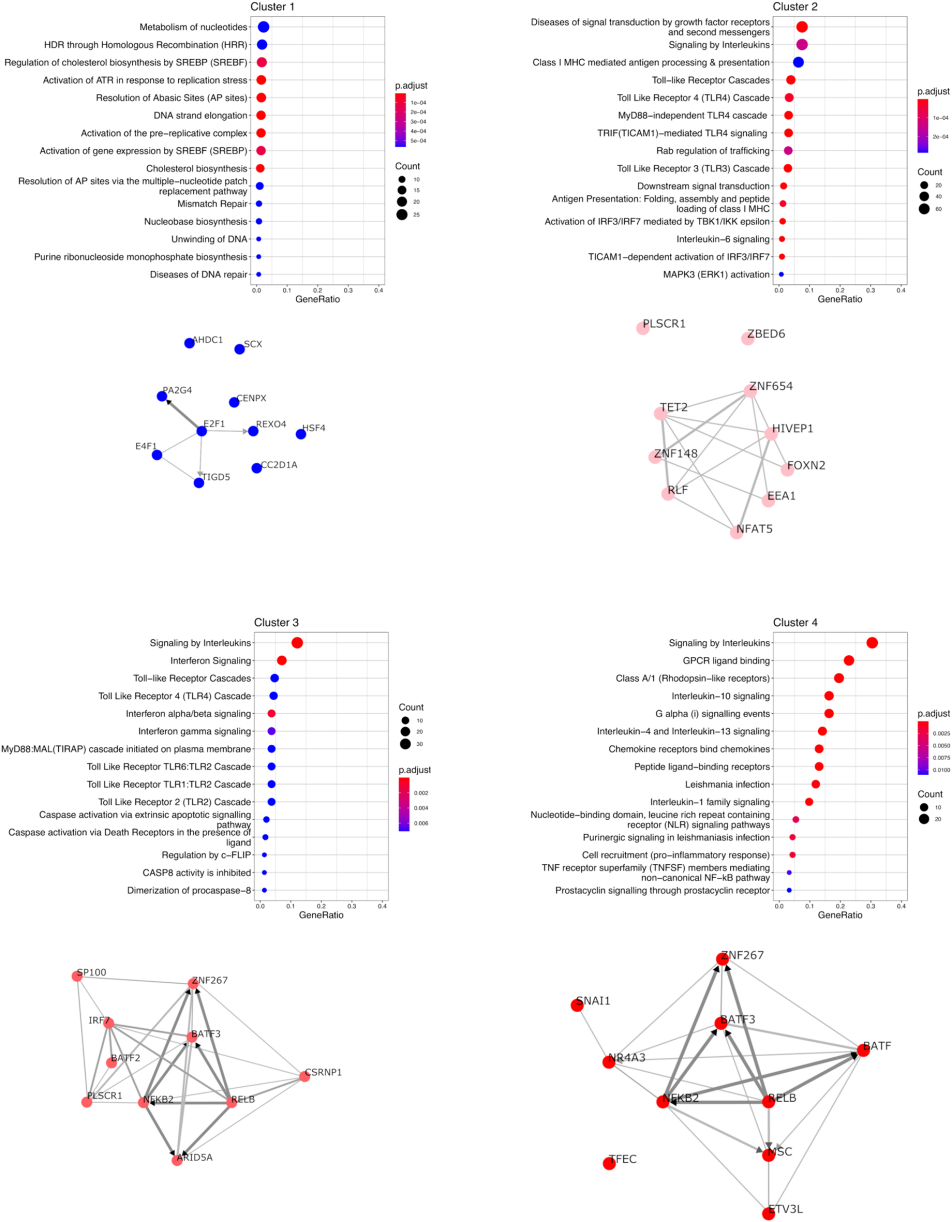
**ReactomePA analysis of the four gene clusters.** pvalueCutoff for ReactomePA was set to 0.01 and the top 15 pathways are represented for each cluster. The *p*-adjust shows the significance of the enrichment of a function within the DEGs, adjusted by Benjamini and Hochberg’s FDR. The size of the dots represents the ratio of DEGs/number of genes in the pathway. The results of CHEA3 analysis for each gene cluster are represented below the ReactomePA plot.

For each of the 4 clusters identified, we performed an analysis of enriched pathways using the Reactome database and a transcription factor (TF) enrichment analysis (TFEA) in order to identify TFs potentially responsible for the observed changes in gene expression (Figure 5). The “Signaling by interleukins” pathway is enriched in both clusters 3 and 4. Clusters 3 and 4 share potential TF candidates, in particular NF-KB related TF (NF-KB2 and RELB) as well as BATF3 and ZNF267. Among the notable results are the enrichment of the “Interferon signaling” pathway in cluster 3 genes, consistent with the identification of IRF7 as a candidate TF involved in the regulation of cluster 3 genes.

### sCD14 is essential for full activation of Myd88-independent pathway by smooth LPS

A comparison of responses triggered by rough (LPSR) and smooth (LPSS) LPS in the absence of sCD14 showed that, in these conditions, a total of 503 genes listed in Additional file 5 were found to be differentially expressed including 12 lncRNA and 1 miRNA (miR-147). Pathway analyses were performed against the KEGG pathway database using SPIA and against the Reactome database using ReactomePA. Volcano plot of these analyses and results of the Reactome analysis are depicted in Additional file 6. These analyses showed the “Cytokine-cytokine receptor interaction” and “Toll-like receptor signaling” pathways and more specifically the “MyD88-independent TLR4 cascade” as being differently regulated between LPSR and LPSS in the absence of sCD14 (Table 1). This first analysis clearly shows that, in the absence of sCD14, the response of PS cells to LPSR is more pro-inflammatory than the one observed with LPSS.

**Table 1:** **Top 10 pathways identified by SPIA as differentially activated between LPSR-NONE and LPSS-NONE stimulated PS cells**

KEGG pathway name	KEGG pathway ID	Size	pNDE	pPERT	pG	pGFdr	Status
Cytokine-cytokine receptor interaction	04060	144	1.23E−16	0.0001	5.75E−19	6.96E−17	Activated
NF-kappa B signaling pathway	04064	74	3.63E−13	0.68	7.41E−12	4.48E−10	Activated
NOD-like receptor signaling pathway	04621	48	2.36E−12	0.72	4.77E−11	1.92E−09	Inhibited
Rheumatoid arthritis	05323	57	3.75E−12	0.8	8.26E−11	2.5E−09	Activated
Chemokine signaling pathway	04062	125	4.86E−08	0.0001	1.32E−10	3.18E−09	Activated
Malaria	05144	33	4.29E−10	0.36	3.64E−09	7.35E−08	Activated
Measles	05162	91	1.47E−09	0.86	2.72E−08	4.57E−07	Inhibited
Osteoclast differentiation	04380	90	8.83E−09	0.16	3.02E−08	4.57E−07	Activated
Toll-like receptor signaling pathway	04620	75	3.81E−09	0.44	3.55E−08	4.78E−07	Inhibited
Chagas disease (American trypanosomiasis)	05142	78	6.97E−09	0.96	1.33E−07	1.6E−06	Activated

The list of DEG were analyzed with the SPIA package to identify, among the pathways of the KEGG database, those that are enriched in the set of DEG between LPSR-NONE and LPSS-NONE. The table indicates the size of the pathway in the KEGG database, the probability of the significance of the given pathway Pi as provided by an over-representation analysis of the number of DEG (pNDE), the probability based on the amount of perturbation measured in each pathway (pPERT). pG is a global probability value combining pNDE and pPERT. pGFdr is an adjusted value of pG for multiple comparisons (pGFdr).

**Figure 6:**
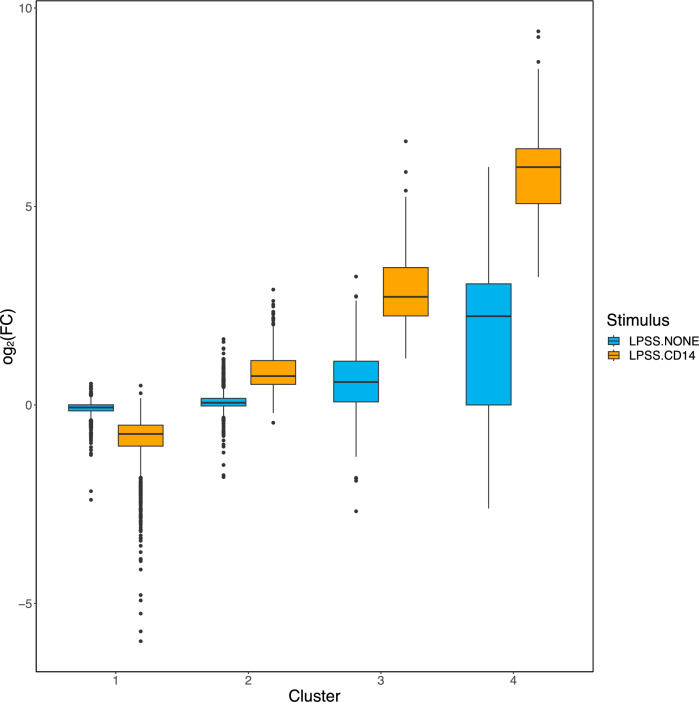
**Box-and-whisker plot of the log**_**2**_**(FC) of genes belonging to each gene cluster between LPSS-CD14 and LPSS-NONE conditions.** The log_2_(FC) for genes belonging to each of the four clusters between LPSS-CD14 (orange) and LPSS-NONE (light blue) conditions were calculated using edgeR and are represented.

Based on the fact that most *E. coli* strains express smooth LPS, along with rough LPS, and that milk contains a significant amount of sCD14, we then focused our analysis on the comparison of transcriptomic profiles obtained upon stimulation of PS cells with LPSS in the presence or absence of sCD14 [16, 27]. The mean log_2_(FC) in these two conditions was calculated for genes of the four clusters identified above (Figure 6). While there are only small differences in cluster 1 and 2 genes, cluster 3 genes are activated by LPSS only in the presence of sCD14: the average log_2_(FC) for cluster 3 genes in the absence of sCD14 was 0.57 ± 0.83 compared to the unstimulated control, with only few significant differences (47/336), while the average log_2_(FC) compared to unstimulated control was 2.17 ± 0.89 in the presence of sCD14, mostly with FDR below 0.01 (197/347). Cluster 4 genes are activated by LPSS, both in the presence (5.96 ± 1.26) or absence, albeit at a lower level (1.99 ± 1.77), of sCD14 compared to unstimulated control.

**Figure 7:**
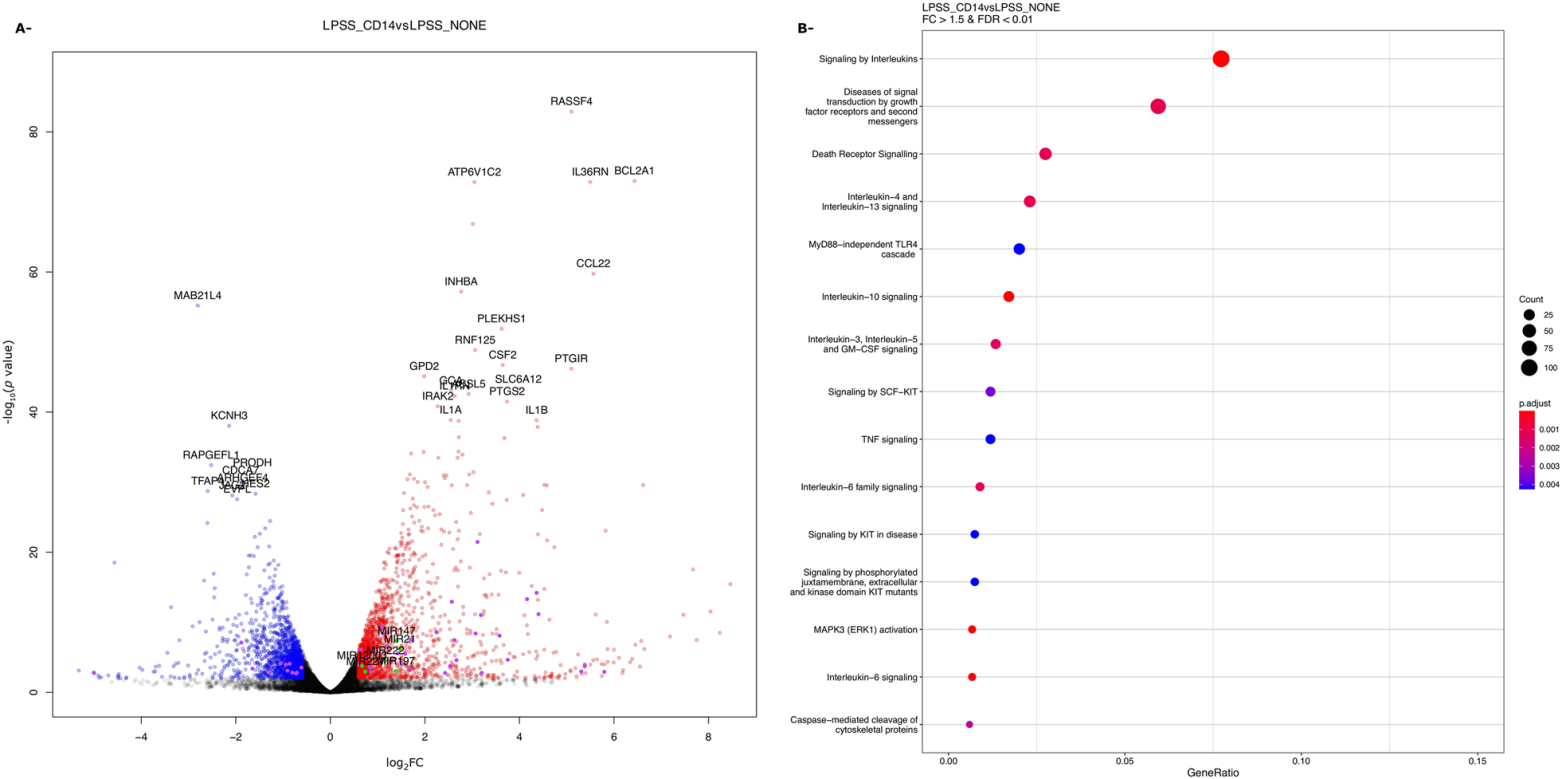
**Volvano plot and ReactomePA analysis of DEG between LPSS-CD14 and LPSS-NONE conditions.** The log_2_(FC) and *p*-values for DEG between LPSS-CD14 and LPSS-NONE conditions are represented as a volcano plot. Red and blue dots indicate genes overexpressed and under-expressed, respectively. Purple and green dots indicate lncRNA and miRNA, respectively.

Comparing LPSS-CD14 vs LPSS-NONE samples, 2527 genes, listed in Additional file 7, were significantly differentially expressed, among which were 47 lncRNA and 6 miRNA (miR-221, miR-21, miR-222, miR-197, miR-147, miR-12003) (Figure 7A). SPIA analysis indicated a number of pathways were upregulated in the presence of sCD14, in particular pathways related to “cytokine-cytokine receptor interaction” and the “Toll-like receptor signaling pathway” (Table 2). A detailed view of the gene expression changes of these two pathways is depicted in Additional files 8 and 9. A number of genes encoding pro-inflammatory cytokines are more expressed by LPSS in the presence of sCD14.

**Table 2:** **Top 10 pathways identified by SPIA as differentially activated in PS cells by LPSS in the presence of CD14 compared to LPSS without CD14**

KEGG pathway name	KEGG pathway ID	Size	pNDE	pPERT	pG	pGFdr	Status
Cytokine-cytokine receptor interaction	04060	144	5.45E−10	0.0001	1.72E-12	2.36E-10	Activated
Chemokine signaling pathway	04062	125	6.99E−06	0.0001	1.54E−08	1.06E−06	Activated
MAPK signaling pathway	04010	203	7.91E−08	0.02	3.37E−08	1.54E−06	Activated
NF-kappa B signaling pathway	04064	74	5.64E−07	0.02	2.18E−07	7.46E−06	Activated
Pathways in cancer	05200	259	4.41E−08	0.48	3.95E−07	1.08E−05	Activated
Amoebiasis	05146	76	3.24E−07	0.14	8.11E−07	1.85E−05	Activated
Toll-like receptor signaling pathway	04620	75	8.04E−07	0.08	1.13E−06	2.21E−05	Activated
Toxoplasmosis	05145	98	1.06E−06	0.2	3.47E−06	5.94E−05	Activated
Jak-STAT signaling pathway	04630	102	1.12E−06	0.3	5.35E−06	8.15E−05	Activated
Osteoclast differentiation	04380	90	2.34E−05	0.02	7.29E−06	9.99E−05	Activated

The list of DEG were analyzed with the SPIA package to identify, among the pathways of the KEGG database, those that are enriched in the set of DEG between LPSS-CD14 and LPSS-NONE. The table indicates the size of the pathway in the KEGG database, the probability of the significance of the given pathway Pi as provided by an over-representation analysis of the number of DEG (pNDE), the probability based on the amount of perturbation measured in each pathway (pPERT). pG is a global probability value combining pNDE and pPERT. pGFdr is an adjusted value of pG for multiple comparisons (pGFdr).

**Figure 8:**
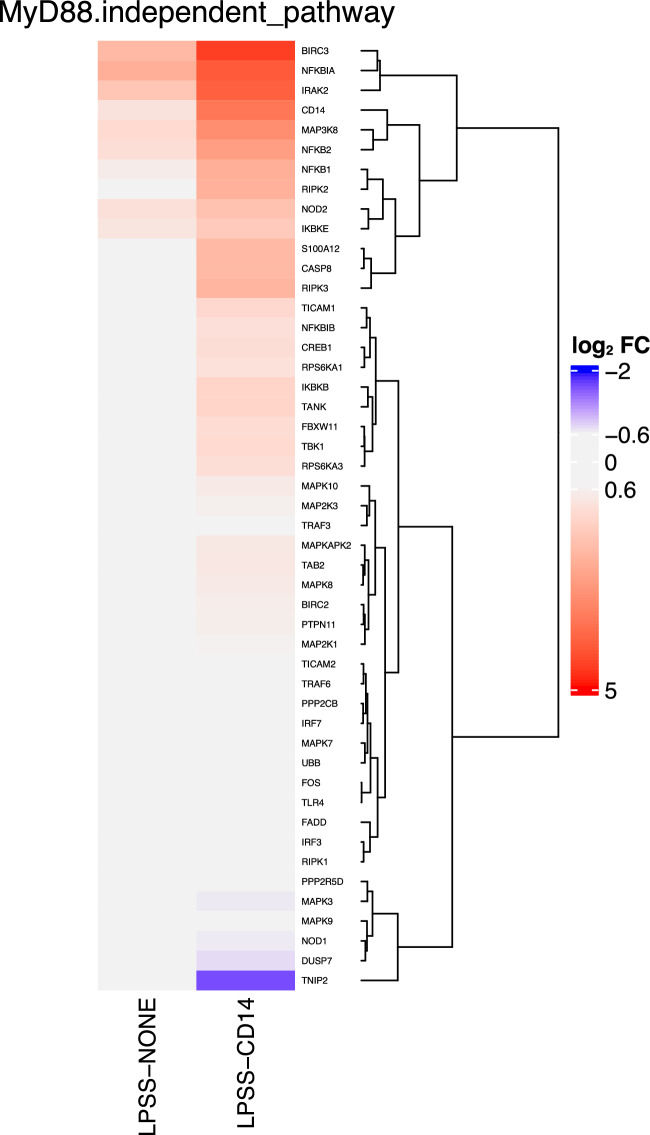
**Heatmap of log**_**2**_**(FC) of genes belonging to the Myd88-independent pathway.** Log_2_(FC) of DEG belonging to the Myd88-independent pathway in conditions LPSS-NONE and LPSS-CD14 compared to the CTRL-NONE samples are represented.

Top signaling pathways identified by ReactomePA are reported in Figure 7B. The main signaling cascades found contain the Signaling by Interleukin pathway, diseases of signal transduction, and the Toll Like Receptor 4 (TLR4) Cascade and in particular the MyD88-independent TLR4 signaling cascade. Among DEGs associated in this last pathway, the majority were up-regulated. A closer investigation of fold-changes of genes belonging to the MyD88-independent TLR4 signaling cascade clearly highlights that this pathway is activated much more strongly by LPSS only in the presence of sCD14 (Figure 8).

Overall, these data confirm the importance of sCD14 as a cofactor for an efficient response of MEC to *E. coli* LPS. The main difference that can be observed when cells are stimulated in the presence of sCD14 is a much higher number of cytokine genes whose expression is induced, which corresponds to the activation of the Myd88-independent TLR4 cascade.

### miRNA and lncRNA genes regulated by stimulation of MEC by LPS

Our analyses evidenced a number of miRNA and genes annotated as lncRNA whose expression is altered upon stimulation of MEC with the different forms of LPS in the presence or absence of sCD14. As mentioned above, only a few miRNA genes could be identified as differentially expressed: this low number might stem from the RNA extraction and processing which was initially designed to have a global overview of the MEC transcription mRNA profile and is very likely to have missed a significant number of small miRNA. Concerning lncRNA, we initially used ENSEMBL annotations and confirmed these loci were indeed lncRNA by using the FEELnc pipeline [28]: this led to a total of 940 FEELnc confirmed lncRNA, out of 1480 annotated lncRNA in the ENSEMBL database.

**Figure 9:**
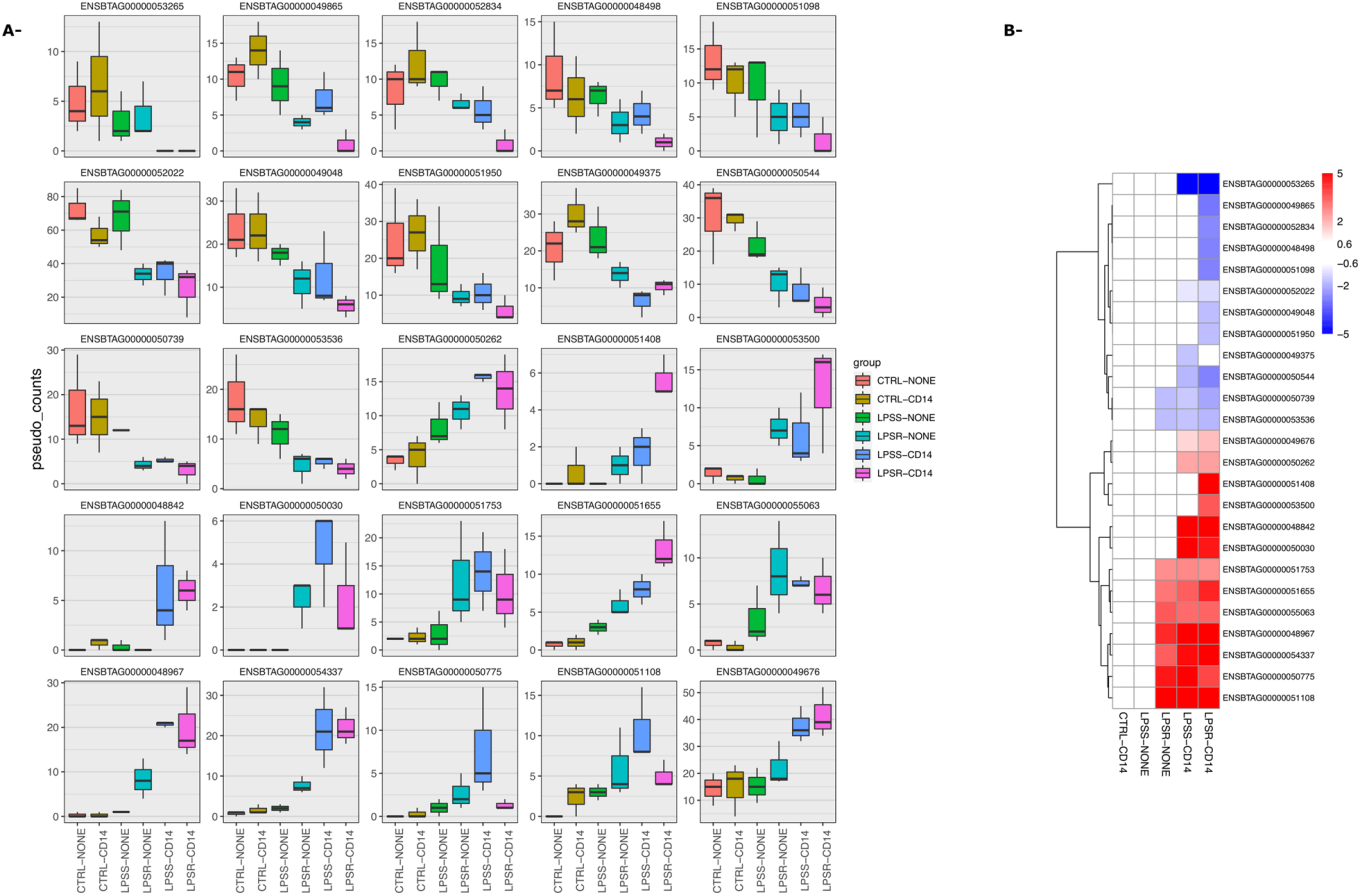
**Expression of lncRNA in the different conditions tested.** Differentially regulated lncRNA in at least one condition were identified using the edgeR package. Expression values are represented for each of these lncRNA as pseudo-count values obtained using the edgeR package.

Among these lncRNA, 25 were significantly differentially expressed compared to the unstimulated cells in at least one condition. Interestingly, among these DEG lncRNA, none of them was significantly differentially expressed in LPSS-NONE samples indicating that sCD14 was strictly required for their deregulation (Figure 9). One of these lncRNA, ENSBTAG00000054337, is located immediately downstream of the mi-RNA let7C-201 that has been involved in the negative regulation of TLR4-mediated LPS response in murine macrophages [29].

## Discussion

Our previous results had already identified sCD14 as a regulator of key chemokines involved in the innate response of bovine mammary epithelial cells. The current report provides a complete overview of the impact of sCD14 on the response of MECs.

Early reports illustrated how sCD14 infusion in vivo could help the bovine mammary gland eliminate *E. coli*. The role of sCD14 is likely not to be restricted to a contribution to the mammary gland defence: the presence of high concentration of sCD14 in milk potentially contributes to the offspring innate immune defence [30, 31].

A key question that remains to be answered is the origin of the sCD14 present in milk at concentrations in the range of 2–3 µg/mL. One hypothesis is the shedding of mCD14 from the milk leucocyte membrane. An alternative hypothesis is the secretion of sCD14 in milk by mammary epithelial cells as has been described in human mammary epithelial cells [30]. Our results clearly indicate that mammary epithelial cells do not express mCD14 but are able to secrete sCD14. The absence of mCD14 on the surface of bovine MEC is consistent with results obtained in mice studies showing that in the absence of inflammation mCD14 is not detected in the mammary gland epithelium [32]. Because the amount of sCD14 is significant even in udder quarters with very low somatic cell counts (< 10 000 cells/mL), it is very likely that most of milk sCD14, in a healthy udder, originates from mammary epithelial cells [16].

Polymorphisms in the bovine CD14 gene have been identified, some of them potentially linked to increased resistance against mastitis [33–35]. Such polymorphisms could have consequences on the activity of CD14 but also, when located in the promoter region for example, on the level of sCD14 in milk as has been observed in women breast milk [36].

In order to more specifically address how sCD14 and full-length LPS (i.e. LPSS) modulate the mammary epithelial cells response, we undertook an RNAseq analysis of the transcriptome of cells from the PS cell line, a mammary epithelial cell line previously described [14]. Cells were stimulated with LPS molecules with or without O-antigen, in the presence or absence of sCD14.

A major function of CD14 is to allow cells to respond to full-length LPS molecules, that is molecules that possess an O-antigen [13]. Indeed, as evidenced by KEGG and Reactome pathways analyses performed in this study, the presence of the O-antigen reduces the pro-inflammatory response of PS cells in the absence of sCD14: a number of genes were significantly more induced by LPSR which results in a more pronounced activation of the Cytokine-cytokine receptor interaction pathway, the NF-kappa B signaling pathway, the Chemokine signaling pathway and the Toll-like receptor signaling pathway, all of which are involved in the innate immune response of the host. Only when sCD14 was provided could a full-scale response of PS cells be obtained.

A global analysis of genes deregulated in at least one of the conditions tested identified four different clusters of genes with different expression patterns. Cluster 1 encompasses genes whose expression is reduced upon stimulation with LPS while the genes in the three other clusters showed induction of expression upon stimulation, the three clusters being differentiated by the level of induction observed. Each cluster was also enriched in genes belonging to different pathways from the REACTOME database. In particular, cluster 3 genes were enriched in genes from the “Interferon signaling” pathway and the IRF7 transcription factor was, among others, identified by CHEA3 as one of the transcription factors for which targets are enriched in cluster 3.

Cluster 3 genes are characterized by a significant induction when PS cells are stimulated with smooth LPS in the presence of sCD14 but not in the absence of sCD14: the detailed comparison of LPSS-CD14 and LPSS-NONE samples identified a large number of DEG with an enrichment for genes of the KEGG “Cytokine-cytokine receptor interaction” pathway. Of note, some receptors, in particular the IFNAR1 and IFNAR2 receptors, are significantly more expressed upon stimulation with LPSS in the presence of sCD14 (Additional file 8). This could allow cells to be more responsive to type I interferons in the mammary gland. Yet, type 1 interferon genes were not expressed by PS cells under any of the conditions tested in this report. Furthermore, secretion of type I interferons in milk from mastitic cows has never been demonstrated. A potential role for type 1 interferon in the innate response of the udder remains to be demonstrated.

In addition to the activation of the interferon signaling by LPSS only in the presence of sCD14, a few key features are worth mentioning. First, we observed that the antigen processing and presentation pathway was activated upon stimulation of PS cells with LPS. In particular, CD40, CD80 and CD83 genes were upregulated by LPSR and LPSS, with or without sCD14 (Additional files 5 and 7). Bovine MECs had been shown to overexpress the co-stimulatory molecule CD83 upon stimulation with LPS [37]. Because these molecules are co-stimulatory molecules for antigen presentation, the potential contribution of activated MECs to antigen presentation in the mammary gland would be worth investigating. The expression of the corresponding proteins and the relevance of this induction during mastitis in vivo should be characterized. The overexpression of co-stimulatory molecules, along with the expression of MHC class II molecules by bovine MECs might contribute to the immune response of sensitized mammary glands to bacterial antigens [38, 39].

Recently, activation of the Nrf2 pathway by LPS in mammary epithelial cells has been suggested [40]. Among the genes investigated by the authors, we observed induction of Nrf2 and HMOX1 but not of NQO1, nor GCLM. A more specific analysis of genes belonging to this pathway annotated in the Reactome database is presented in Additional file 10 and shows induction of only some of the associated genes.

Expression of lncRNA upon LPS stimulation has recently been studied in MAC-T cells, another mammary epithelial cell line [41]. Unfortunately, we were not able to confirm the results presented in [41] with the one described herein. A potential explanation is differences in annotations since this report used a previous annotation that contained only 210 lncRNA and 2000 new lncRNA not annotated in ENSEMBL.

Among the lncRNA which we identified as differentially expressed, one was located in the close vicinity of the let-7c-201 miRNA. The relevance of this lncRNA is illustrated in Additional file 11 showing that it is partly conserved in the human genome, the first 519 nucleotide of ENSBTAG00000054337 showing 88% identity at the DNA level with the 5’ region of the Homo sapiens mir-99a-let-7c cluster host gene (MIR99AHG). Interestingly, this lncRNA has been identified as a positive regulator of inflammation and macrophage polarization to promote growth of *Mycobacterium tuberculosis* [42]. Whether this lncRNA plays a role in the response of MEC to *E. coli* infection will have to be deciphered in more details.

Altogether, our results demonstrate that the presence of sCD14 is required for a full-scale response of MEC to stimulation by LPSS. O-antigen is present on most, if not all, *E. coli* strains causing mastitis as its absence causes strains to be susceptible to complement mediated lysis in milk and thus unable to grow in milk. The presence of sCD14 is therefore an essential component of the response of the mammary gland to coliforms, allowing activation of different pathways involved in triggering the host pro-inflammatory response.

### Supplementary Information

**Additional file 1. bioinformatic tools used in RNAseq analyses.**

**Additional file 2. Gating strategy used for flow-cytometry analyses.** Primary MEC from three cows and PS cells were labeled with antibodies directed against CD45 and CD14 and with a viability marker (Fixable Viability dye eFluor 450). Single cells (“Mono”) were selected on the FSC-A/FSC-H plot. Debris were then excluded and cells were selected on the FSC-A/SSC-A plot (“Cells”). Live cells were then selected based on eFluor 450 staining (“Live cells). Thresholds for CD14 and CD45 labeling were set based on non-labelled cells and control labeling on PBMC.

**Additional file 3. MDS plot and correlation plots performed on the 15,700 DEGs in response to LPS and/or CD14 contact.** (A) RNAseq data were analyzed with the edgeR package. Weakly expressed genes, with less than 5 reads in all samples, were excluded from the analysis. Multi-dimensional (MDS) plot was generated with the plotMDS function and illustrates the distribution of samples in a 2-dimensions plot. The gray, black, light blue, blue, orange, and red colors indicate samples from the conditions CTRL-NONE, CTRL-CD14, LPSS-NONE, LPSR-NONE, LPSS-CD14 and LPSR-CD14, respectively. The numbers -1, -2 or -3 in the name of the samples represent the number of the experiment. The distances between samples correspond to the biological coefficient of variation (BCV). (B) Correlation plot was based on the expression values (log_2_(pseudo_counts)) obtained by the edgeR package of only differentially expressed genes (abs(log_2_(FC) > 0.6) and *p*-value > 0.05). The top-left color key indicates the correspondence between colors and value of the correlation value between samples.

**Additional file 4. Heatmap of log**_**2**_**(FC) of genes differentially regulated miRNA.** Log_2_(FC) of DEG enconding miRNA in conditions CTRL-CD14, LPSS-NONE, LPSR-NONE, LPSS-CD14 and LPSR-CD14 compared to the CTRL-NONE samples are represented.

**Additional file 5. list of genes differentially expressed between conditions LPSR-NONE and LPSS-NONE.** DEG between conditions LPSR-NONE and LPSS-NONE were identified using the edgeR package. A gene was considered differentially expressed when its expression was increased or decreased by a factor of 1.5 (absolute value of fold-change > 1.5) with a false-discovery rate (FDR) of 0.01. In the table below, the log_2_ of the FC is indicated as well as the *p*-value and FDR. Genes are identified by their unique ENSEMBL identifier with their corresponding official gene SYMBOL and their ENTREZID reference in the NCBI database.

**Additional file 6. Volvano plot and ReactomePA analysis of DEG between LPSR-NONE and LPSS-NONE conditions.** (A) The log_2_(FC) and *p*-values for DEG between LPSS-CD14 and LPSS-NONE conditions are represented as a volcano plot. Red and blue dots indicate genes overexpressed and under-expressed, respectively. Purple and green dots indicate lncRNA and miRNA, respectively. (B) Results from pathway analysis with the Reactome PA package are represented. pvalueCutoff for ReactomePA was set to 0.01 and the top 15 pathways are represented for each cluster. The p-adjust shows the significance of the enrichment of a function within the DEGs, adjusted by Benjamini and Hochberg’s FDR. The size of the dots represents the ratio of DEGs/number of genes in the pathway.

**Additional file 7. List of genes differentially expressed between conditions LPSS-CD14 and LPSS-NONE.** DEG between conditions LPSS-CD14 and LPSS-NONE were identified using the edgeR package. A gene was considered differentially expressed when its expression was increased or decreased by a factor of 1.5 (absolute value of fold-change > 1.5) with a false-discovery rate (FDR) of 0.01. In the table below, the log_2_ of the FC is indicated as well as the *p-*value and FDR. Genes are identified by their unique ENSEMBL identifier with their corresponding official gene SYMBOL and their ENTREZID reference in the NCBI database.

**Additional file 8. KEGG Cytokine-cytokine receptor interaction genes differentially expressed between LPSS-CD14 and LPSS-NONE conditions.** The “Cytokine-cytokine receptor interaction” pathway was retrieved from the KEGG database using R packages GAGE and Pathview. Boxes corresponding to genes differentially regulated are colored depending of the log_2_(FC) value as indicated by the scale in the top right corner.

**Additional file 9. LPSS.CD14vsLPSS.NONE: KEGG Toll-like receptor signaling pathway genes differentially expressed between LPSS-CD14 and LPSS-NONE conditions.** The “Toll-like receptor signaling pathway” was retrieved from the KEGG database using R packages GAGE and Pathview. Boxes corresponding to genes differentially regulated are colored depending of the log_2_(FC) value as indicated by the scale in the top right corner.

**Additional file 10. Expression of Nrf2-pathway genes in the different conditions tested.** Expression of Nrf2-pathway genes in the different conditions tested are represented, as well as a heatmap representing the log_2_(FC) of genes belonging to the Nrf2 pathway (identified as KEAP1-NFE2L2 pathway in the Reactome database).

**Additional file 11. Alignment of the bovine lncRNA ENSBTAT00000082732 transcript (ENSBTAG00000054337 locus) with the human MIR99AHG gene.** The sequence of the bovine ENSBTAT00000082732 lncRNA was aligned with the MIR99AHG RNA using Geneious software. The top line is a scale in base pairs. The identity panel represents the percentage of identity between the two sequences with colors from green (100% identity) to red (< 20% identity).

## Data Availability

RNAseq data have been deposited at the ENA with the project accession PRJEB61513.

## Abbreviations

DEG: differentially expressed genes
FC: fold-change
FDR: false discovery rate
LPS: lipopolysaccharide
LPSS: smooth lipopolysaccharide
LPSR: rough lipopolysaccharide
MDS: multidimensional scaling
MEC: mammary epithelial cells
TF: transcription factor
