# Supplementary material for: Soluble CD14 produced by bovine mammary epithelial cells modulates their response to full length LPS
Source: Vet Res. 2024 Jun 12;55:76. doi: 10.1186/s13567-024-01329-3 (PMC11170775; doi:10.1186/s13567-024-01329-3)
Supplement: Supplementary file 1 — Additional file 1. bioinformatic tools used in RNAseq analyses. [file 13567_2024_1329_MOESM1_ESM.docx]

**Additional file 1: bioinformatic tools used in RNAseq analyses**

| Software | Version |
| --- | --- |
| nf-core/rnaseq | v1.4.2 |
| Nextflow | v20.01.0 |
| FastQC | v0.11.8 |
| Cutadapt | v2.5 |
| Trim Galore! | v0.6.4 |
| SortMeRNA | v2.1b |
| STAR | vSTAR_2.6.1d |
| HISAT2 | v2.1.0 |
| Picard MarkDuplicates | v2.21.1 |
| Samtools | v1.9 |
| featureCounts | v1.6.4 |
| Salmon | v0.14.1 |
| StringTie | v2.0 |
| Preseq | v2.0.3 |
| deepTools | v3.3.1 |
| RSeQC | v3.0.1 |
| dupRadar | v1.14.0 |
| edgeR | v3.26.5 |
| Qualimap | v2.2.2-dev |
| MultiQC | v1.7 |
