# Supplementary figures and images for: Soluble CD14 produced by bovine mammary epithelial cells modulates their response to full length LPS
Source: Vet Res. 2024 Jun 12;55:76. doi: 10.1186/s13567-024-01329-3 (PMC11170775; doi:10.1186/s13567-024-01329-3)

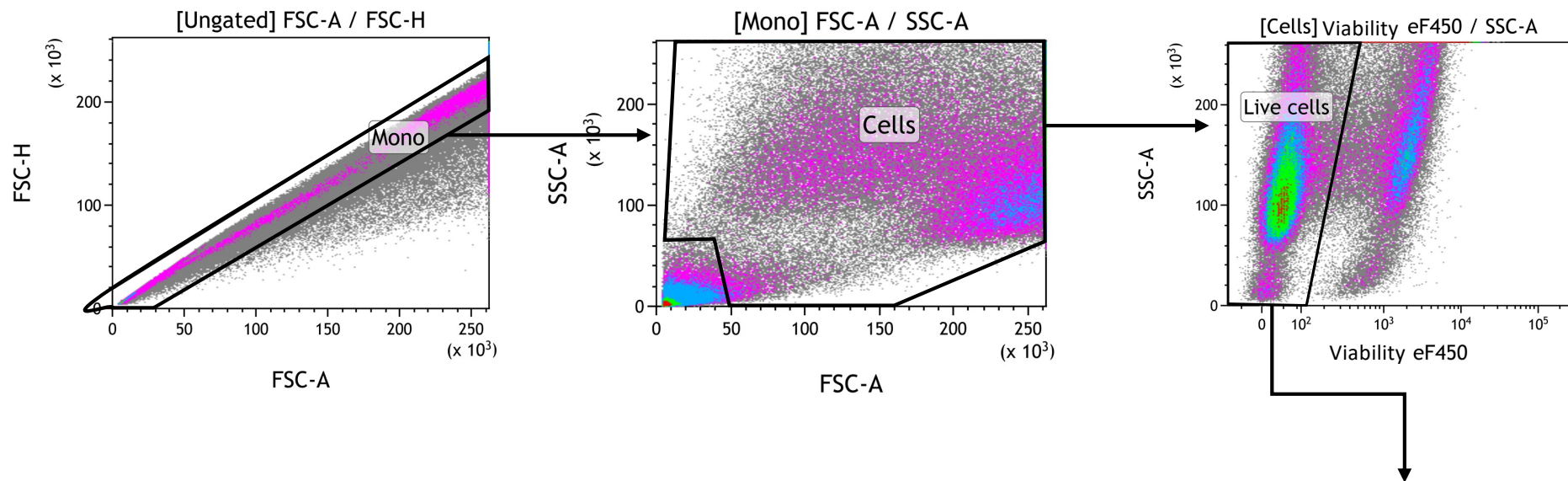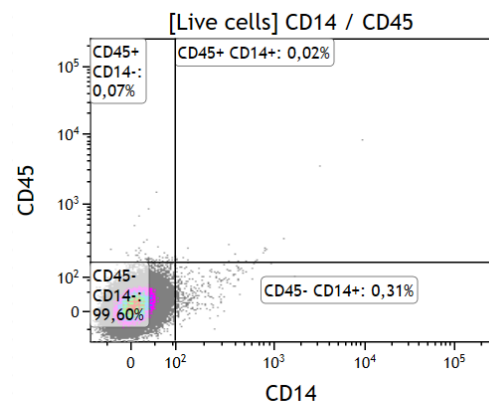

| Gate        | Number  | %Total | %Gated |
|-------------|---------|--------|--------|
| All         | 101 005 | 25,25  | 100,00 |
| CD45- CD14- | 100 598 | 25,15  | 99,60  |
| CD45- CD14+ | 317     | 0,08   | 0,31   |
| CD45+ CD14- | 68      | 0,02   | 0,07   |
| CD45+ CD14+ | 22      | 0,01   | 0,02   |

Supplement: Supplementary file 2 — Additional file 2. Gating strategy used for flow-cytometry analyses. Primary MEC from three cows and PS cells were labeled with antibodies directed against CD45 and CD14 and with a viability marker (Fixable Viability dye eFluor 450). Single cells (“Mono”) were selected on the FSC-A/FSC-H plot. Debris were then excluded and cells were selected on the FSC-A/SSC-A plot (“Cells”). Live cells were then selected based on eFluor 450 staining (“Live cells). Thresholds for CD14 and CD45 labeling were set based on non-labelled cells and control labeling on PBMC. [file 13567_2024_1329_MOESM2_ESM.pdf]

A-

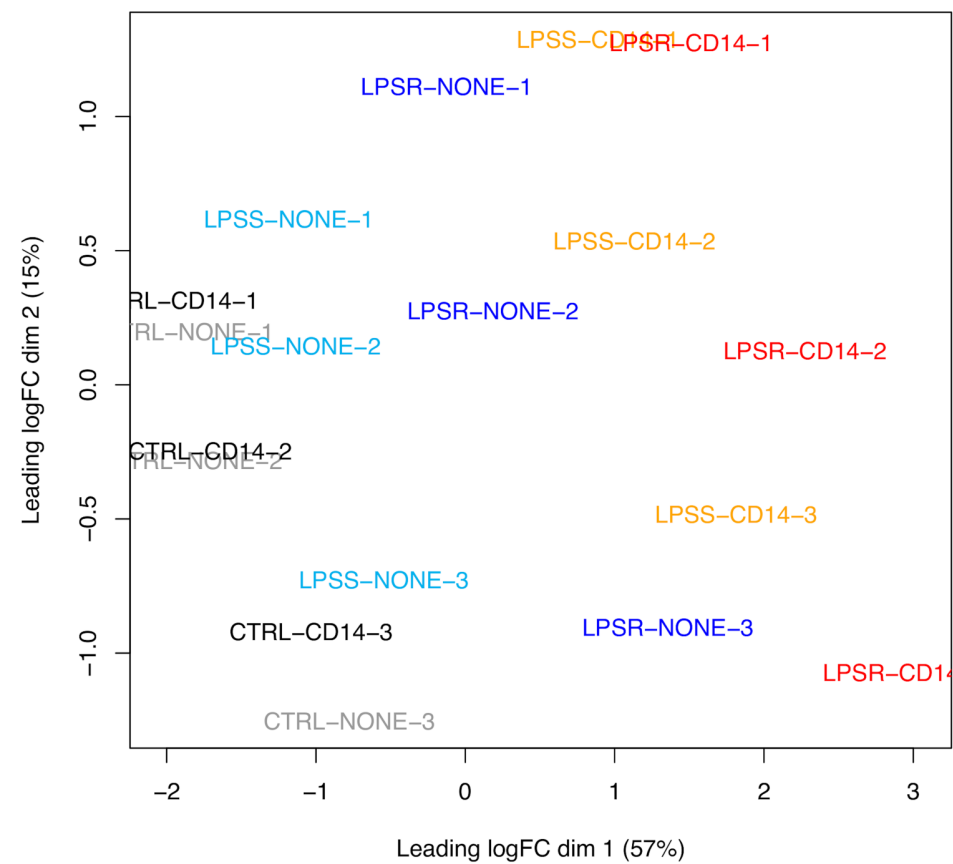

B-

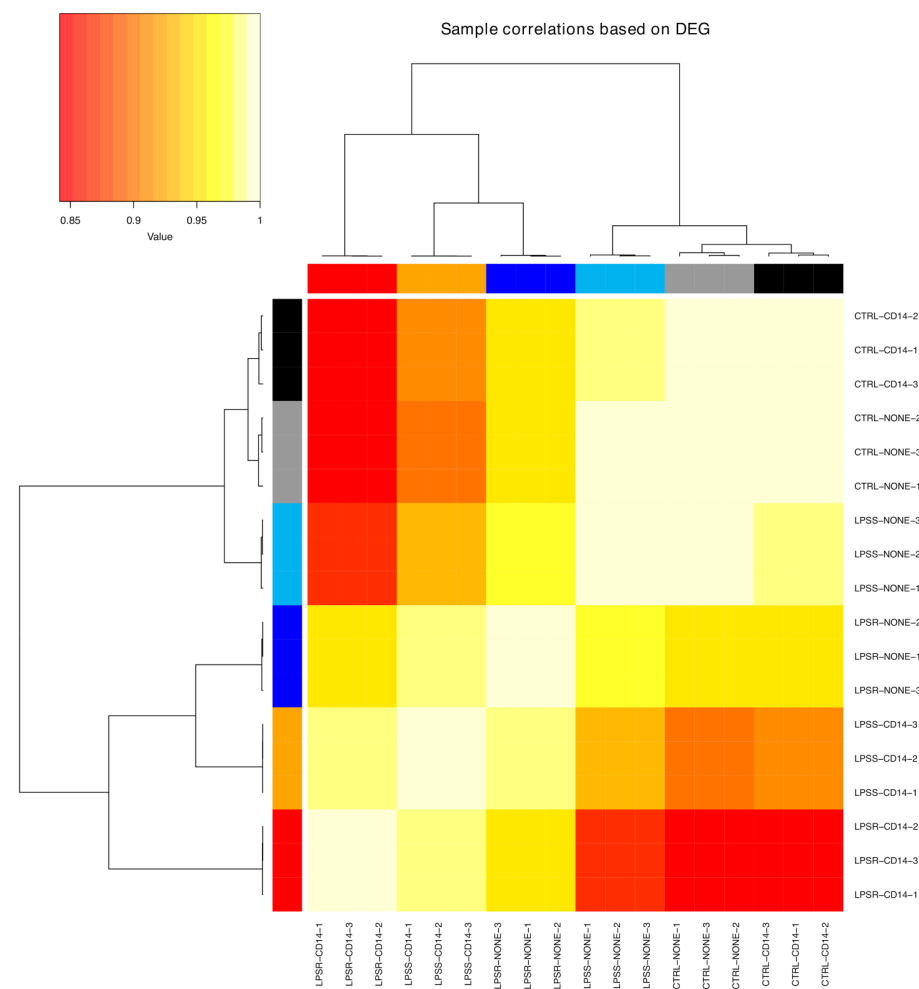

Supplement: Supplementary file 3 — Additional file 3. MDS plot and correlation plots performed on the 15,700 DEGs in response to LPS and/or CD14 contact. (A) RNAseq data were analyzed with the edgeR package. Weakly expressed genes, with less than 5 reads in all samples, were excluded from the analysis. Multi-dimensional (MDS) plot was generated with the plotMDS function and illustrates the distribution of samples in a 2-dimensions plot. The gray, black, light blue, blue, orange, and red colors indicate samples from the conditions CTRL-NONE, CTRL-CD14, LPSS-NONE, LPSR-NONE, LPSS-CD14 and LPSR-CD14, respectively. The numbers -1, -2 or -3 in the name of the samples represent the number of the experiment. The distances between samples correspond to the biological coefficient of variation (BCV). (B) Correlation plot was based on the expression values (log2(pseudo_counts)) obtained by the edgeR package of only differentially expressed genes (abs(log2(FC) > 0.6) and p-value > 0.05). The top-left color key indicates the correspondence between colors and value of the correlation value between samples. [file 13567_2024_1329_MOESM3_ESM.pdf]

DEG miRNA only heatmap  
distance=euclidean – method=ward.D2

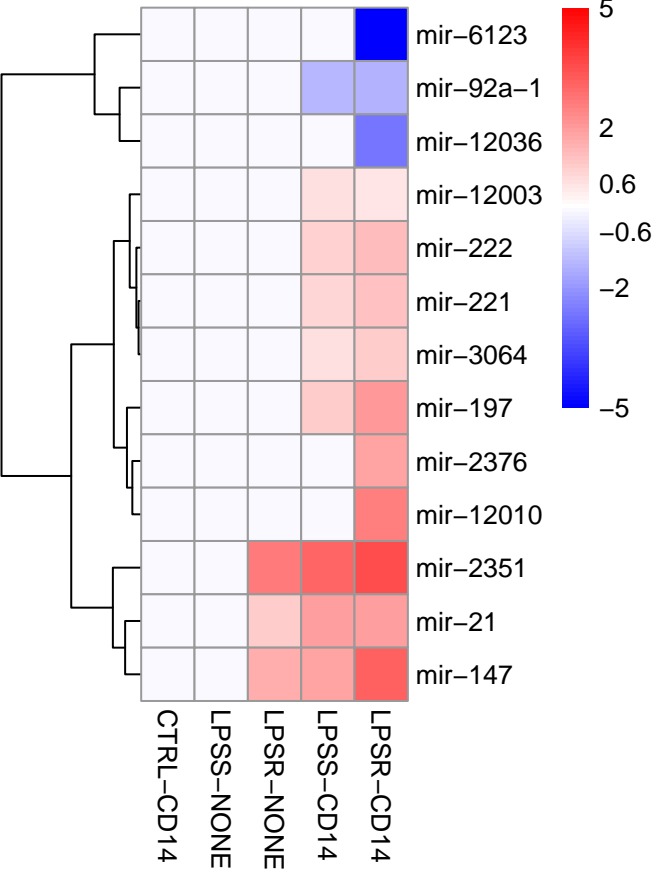

Supplement: Supplementary file 4 — Additional file 4. Heatmap of log2(FC) of genes differentially regulated miRNA. Log2(FC) of DEG enconding miRNA in conditions CTRL-CD14, LPSS-NONE, LPSR-NONE, LPSS-CD14 and LPSR-CD14 compared to the CTRL-NONE samples are represented. [file 13567_2024_1329_MOESM4_ESM.pdf]

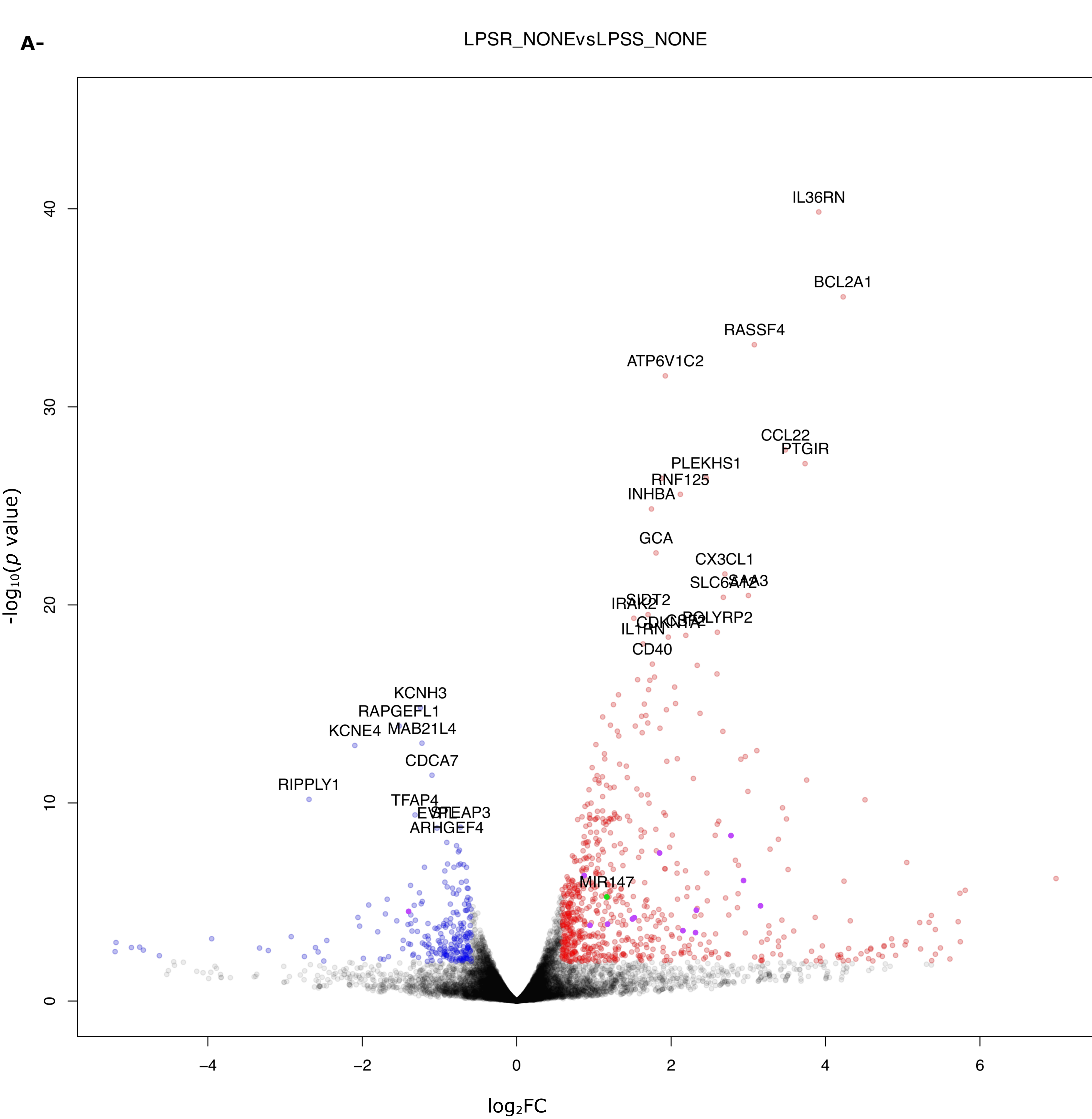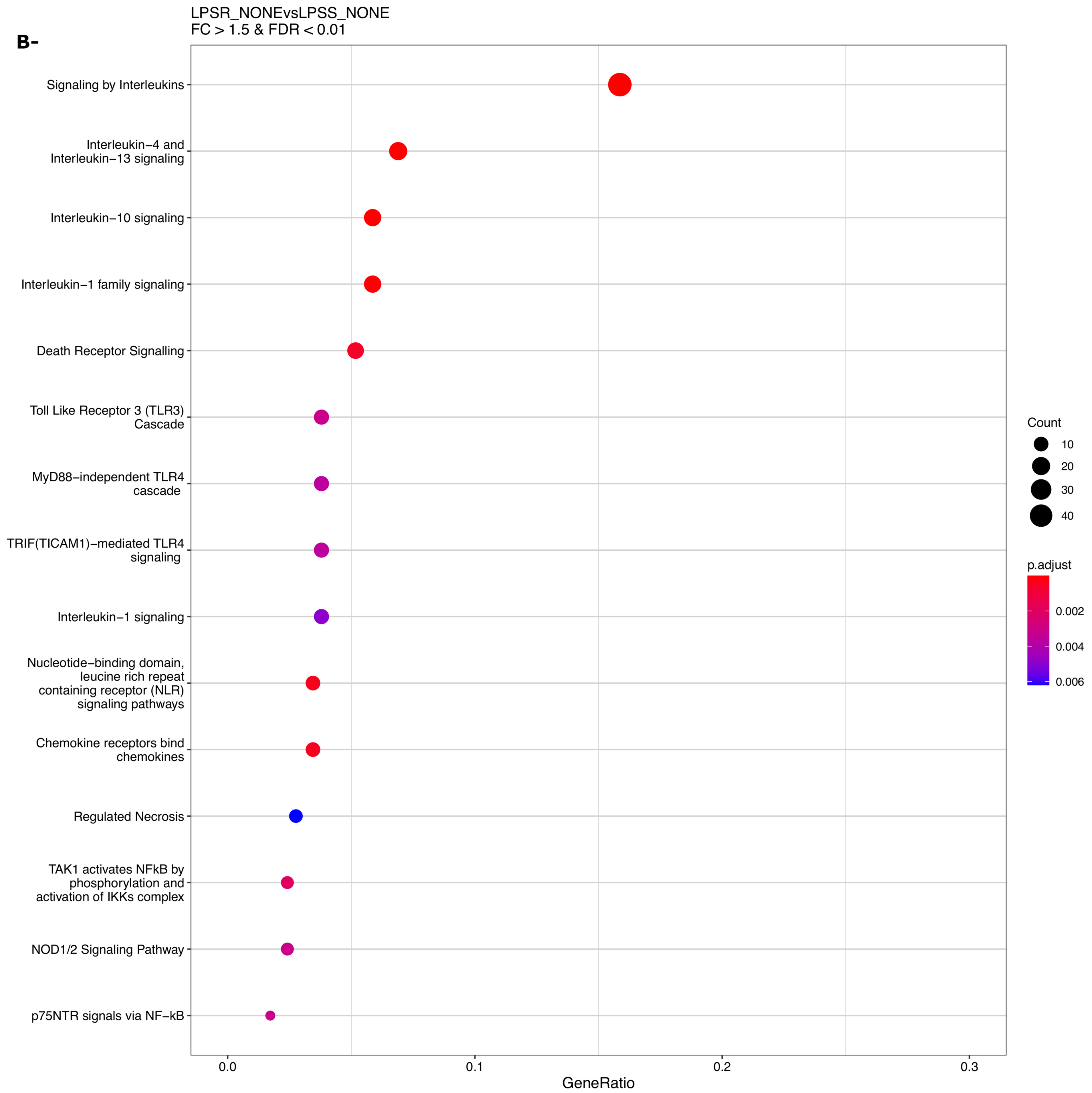

Supplement: Supplementary file 6 — Additional file 6. Volvano plot and ReactomePA analysis of DEG between LPSR-NONE and LPSS-NONE conditions. (A) The log2(FC) and p-values for DEG between LPSS-CD14 and LPSS-NONE conditions are represented as a volcano plot. Red and blue dots indicate genes overexpressed and under-expressed, respectively. Purple and green dots indicate lncRNA and miRNA, respectively. (B) Results from pathway analysis with the Reactome PA package are represented. pvalueCutoff for ReactomePA was set to 0.01 and the top 15 pathways are represented for each cluster. The p-adjust shows the significance of the enrichment of a function within the DEGs, adjusted by Benjamini and Hochberg’s FDR. The size of the dots represents the ratio of DEGs/number of genes in the pathway. [file 13567_2024_1329_MOESM6_ESM.pdf]

# TOLL-LIKE RECEPTOR SIGNALING PATHWAY

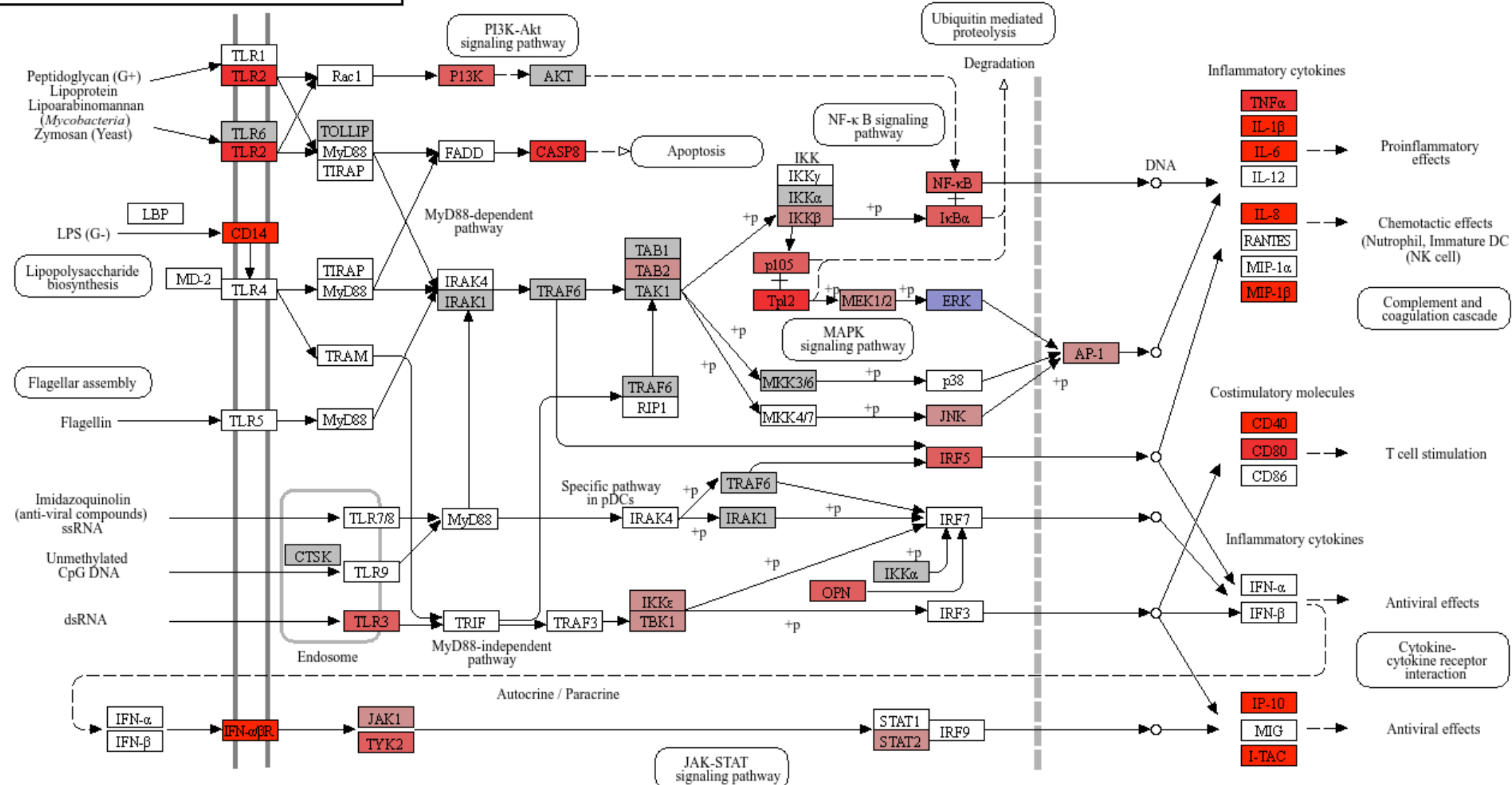

Supplement: Supplementary file 9 — Additional file 9. LPSS.CD14vsLPSS.NONE: KEGG Toll-like receptor signaling pathway genes differentially expressed between LPSS-CD14 and LPSS-NONE conditions. The “Toll-like receptor signaling pathway” was retrieved from the KEGG database using R packages GAGE and Pathview. Boxes corresponding to genes differentially regulated are colored depending of the log2(FC) value as indicated by the scale in the top right corner. [file 13567_2024_1329_MOESM9_ESM.pdf]

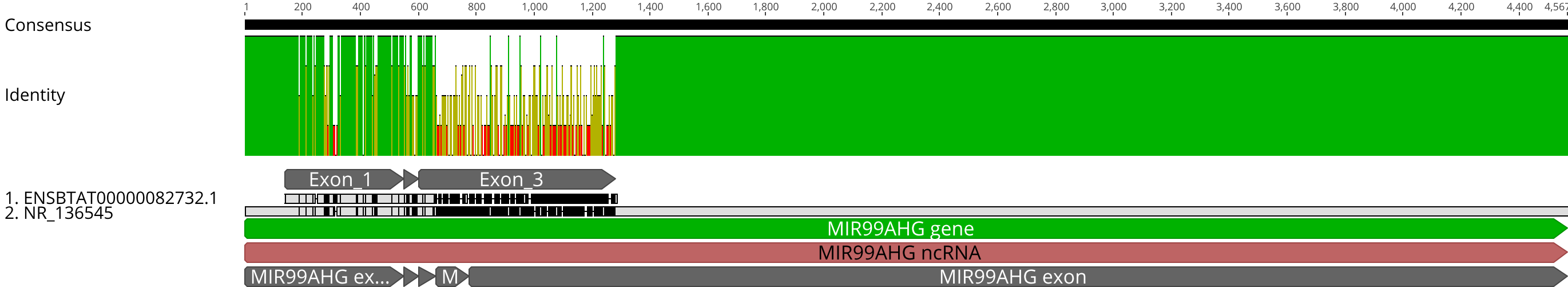

Supplement: Supplementary file 11 — Additional file 11. Alignment of the bovine lncRNA ENSBTAT00000082732 transcript (ENSBTAG00000054337 locus) with the human MIR99AHG gene. The sequence of the bovine ENSBTAT00000082732 lncRNA was aligned with the MIR99AHG RNA using Geneious software. The top line is a scale in base pairs. The identity panel represents the percentage of identity between the two sequences with colors from green (100% identity) to red (< 20% identity). [file 13567_2024_1329_MOESM11_ESM.pdf]
