# Supplementary material for: Soluble CD14 produced by bovine mammary epithelial cells modulates their response to full length LPS
Source: Vet Res. 2024 Jun 12;55:76. doi: 10.1186/s13567-024-01329-3 (PMC11170775; doi:10.1186/s13567-024-01329-3)
Supplement: Supplementary file 8 — Additional file 8. KEGG Cytokine-cytokine receptor interaction genes differentially expressed between LPSS-CD14 and LPSS-NONE conditions. The “Cytokine-cytokine receptor interaction” pathway was retrieved from the KEGG database using R packages GAGE and Pathview. Boxes corresponding to genes differentially regulated are colored depending of the log2(FC) value as indicated by the scale in the top right corner. [file 13567_2024_1329_MOESM8_ESM.pdf]

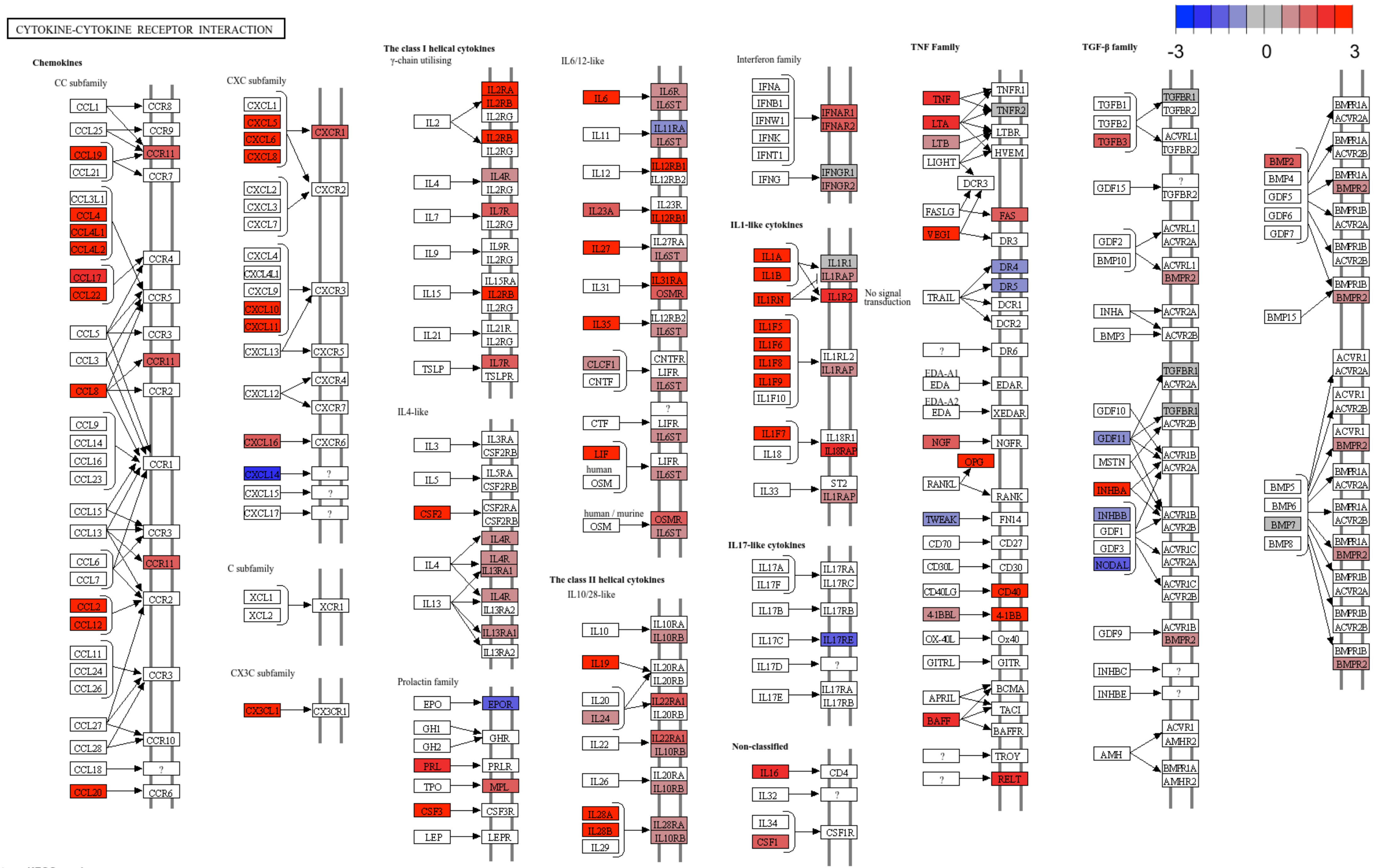

Data on KEGG graph
Rendered by Pathview
