# Supplementary material for: Soluble CD14 produced by bovine mammary epithelial cells modulates their response to full length LPS
Source: Vet Res. 2024 Jun 12;55:76. doi: 10.1186/s13567-024-01329-3 (PMC11170775; doi:10.1186/s13567-024-01329-3)
Supplement: Supplementary file 10 — Additional file 10. Expression of Nrf2-pathway genes in the different conditions tested. Expression of Nrf2-pathway genes in the different conditions tested are represented, as well as a heatmap representing the log2(FC) of genes belonging to the Nrf2 pathway (identified as KEAP1-NFE2L2 pathway in the Reactome database). [file 13567_2024_1329_MOESM10_ESM.pdf]

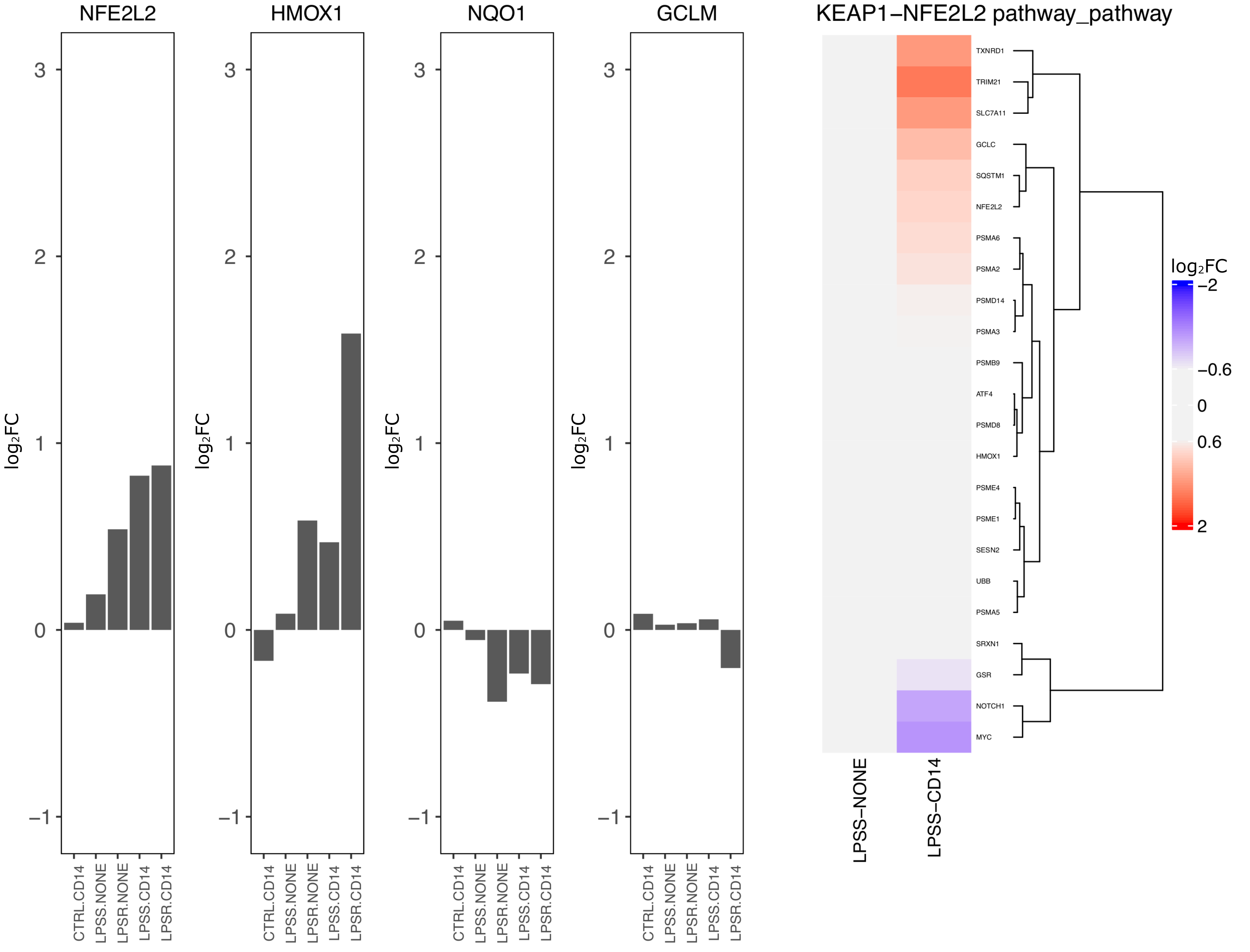

NFE2L2
HMOX1
NQO1
GCLM
KEAP1−NFE2L2 pathway_pathway
log₂FC
3
2
1
0
−1
CTRL.CD14
LPSS.NONE
LPSR.NONE
LPSS.CD14
LPSR.CD14
TXNRD1
TRIM21
SLC7A11
GCLC
SQSTM1
NFE2L2
PSMA6
PSMA2
PSMD14
PSMA3
PSMB9
ATF4
PSMD8
HMOX1
PSME4
PSME1
SESN2
UBB
PSMA5
SRXN1
GSR
NOTCH1
MYC
LPSS−NONE
LPSS−CD14
log₂FC
−2
−0.6
0
0.6
2
